# The Effect of a Regular Auditory Context on Perceived Interval Duration

**DOI:** 10.3389/fpsyg.2018.01567

**Published:** 2018-09-11

**Authors:** Silvia Zeni, Nicholas P. Holmes

**Affiliations:** School of Psychology, University of Nottingham, Nottingham, United Kingdom

**Keywords:** duration perception, time perception, rhythm, temporal regularity, audition, attention

## Abstract

In the auditory domain, the perceived duration of time intervals is influenced by background sounds – the auditory context in which the intervals are embedded – even when the background may be ignored. Previous research has shown that a regular context made of evenly spaced sounds improves participants’ discrimination of intervals close in duration to the context intervals. These results have been explained in terms of attention and anticipation. The present study reconsiders the effect of context regularity, focusing on the relationships among the intervals in the context and the interval to be estimated. The influence of a regular compared to a non-regular auditory context on interval discrimination was examined with a two interval forced choice task, which required participants to discriminate between the durations of two time intervals. Duration perception was more precise when the intervals to be discriminated were preceded by a regular compared to a non-regular context. This effect of the regular context, however, was not selective for the duration of the first interval to be estimated, contrary to suggestions based on previous evidence.

## Introduction

The ability to perceive the duration of events in the range of milliseconds to seconds is thought to be fundamental to a number of cognitive abilities, including moving under temporal constraints, speech comprehension, music perception, and music production (Buhusi and Meck, 2005). Duration perception, however, is not always veridical or reliable. It is influenced by auditory context - the presence of irrelevant sounds preceding, overlapping with, or succeeding the target sounds (Large and Jones, 1999; Barnes and Jones, 2000). For example, empty intervals delimited by a marker at the beginning and the end are perceived to last longer than both filled intervals, in which the signal to be judged is continuous, and intervals filled by multiple regularly spaced markers (Buffardi, 1971; Thomas and Brown, 1974; Adams, 1977; Rammsayer and Lima, 1991; Rammsayer and Leutner, 1996). Moreover, intervals interspersed with regularly spaced fillers are perceived as longer than intervals with irregularly spaced fillers (Thomas and Brown, 1974). Interestingly, such distortions are not observed when non-temporal properties of the fillers (e.g., the sounds’ amplitudes, or frequencies) are made irregular (Horr and Di Luca, 2015).

Other studies have examined the effect of contextual information on the perception of empty intervals and have shown that duration perception measured in a two interval discrimination task was distorted when an interval of short duration preceded the interval to be discriminated, compared to a condition in which a similar distractor was not present (Karmarkar and Buonomano, 2007; Spencer et al., 2009; Burr et al., 2013).

The auditory context has also been shown to systematically influence the perceived duration of single auditory events embedded in the context (Large and Jones, 1999; Barnes and Jones, 2000). This effect is likely automatic, since it has been observed even when participants were explicitly told to ignore the context (Drake and Botte, 1993; McAuley and Kidd, 1998; Barnes and Jones, 2000). When presented with sequences of evenly spaced sounds, listeners were more accurate in judging the duration of tones that end at expected rather than at unexpected points in time (Large and Jones, 1999; Barnes and Jones, 2000). Accuracy in this task followed an inverted U-shaped profile, with the highest accuracy centered on the expected end points, diminishing for sounds presented earlier or later (Large and Jones, 1999). These results provided the grounds of the dynamic attending theory (DAT), which hypothesizes that attention fluctuates periodically over time, and synchronizes with the periodicity of the ongoing auditory stimulation (Barnes and Jones, 2000). In support of DAT, a regular sequence has also been shown to affect judgments of non-temporal stimulus properties, and the typical U-shaped “expectancy profile” has been observed with a pitch identification task (Jones et al., 2002).

In the present work, the effect of a regular auditory context is re-examined with the intent first to replicate the results of Barnes and Jones (2000), and second to extend them, by focusing on the temporal relationship between the intervals in the context and the first interval to be discriminated, the “reference” interval. The reference interval could either be 100, 70, or 50% of the duration of intervals presented in the context. According to the DAT, duration discrimination should benefit maximally from a regular context when the reference interval is equal in duration to the context interval, due to the fact that attention is at its peak when the end marker for that interval is presented. Discrimination of intervals shorter or longer than the context interval, instead, should progressively decrease. In accordance with DAT, we expected to observe the best discrimination with a reference that is 100% of the context interval. Our second prediction is neither part of the DAT nor in contrast with it: a greater effect should be observed with a 50%, compared to a 70% interval, due to the fact that a 50%, unlike a 70% interval, divides the period into two halves, and demarcates an integer subdivision of the rhythmic context. This is despite the fact that a 50%, relative to a 70% interval, is farther from the context interval in terms of absolute duration.

In our first experiment, we examined the extent to which a regular compared to a non-regular context influences the perceived duration of time intervals. We first examined whether discrimination varied across reference intervals of different duration. Second, we examined to what extent any difference in discrimination with regular compared to non-regular context depended on the relationship among the intervals in the context and the first interval to be estimated. More specifically, we examined whether discrimination was best when the interval to be estimated was equal in duration to the context intervals, thereby replicating the experiment of Barnes and Jones (2000).

## Experiment 1

### Materials and Methods

#### Participants

Twenty-eight participants were recruited. Four were excluded (see below), leaving a sample of 24 participants (mean ±*SD* age = 22.9 ± 3.0 years, 19 female). Participants provided written informed consent and participated in return for payment (£15). All participants reported normal hearing and no history of audiological or neurological disorder. The study was approved by the research ethics committee of the University of Nottingham (SoPEC 932) and was conducted in accordance with the Declaration of Helsinki (as of 2008).

#### Apparatus

Auditory stimuli were presented binaurally with PsychPortAudio (Psychtoolbox v. 3, Brainard, 1997; Pelli, 1997) from a MacBook Pro running MacOS X version 10.9.5 using the built-in Core Audio device and through headphones Philips O’Neill SHO9565BK. All stimuli were digitally created with MATLAB (MathWorks, Natick, MA, United States) at a sampling rate of 44.1 kHz and 16 bit resolution. Responses were collected with a standard USB computer keyboard. The python package Psignifit 3.0 (Fründ et al., 2011) was used for curve fitting; the free software R (R Core Team, 2016) was used for data analysis.

#### Stimuli

**FIGURE 1:**
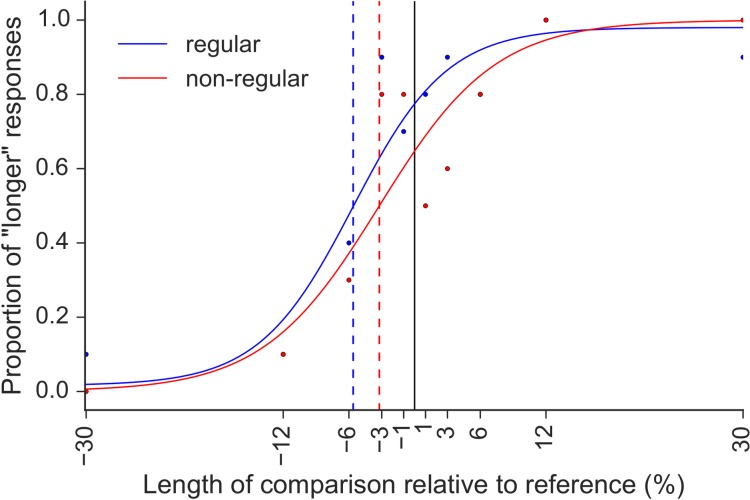
Example of the psychometric functions fitted in Experiment 1 for participant S9 and 660 ms reference, with regular (blue) and non-regular (red) auditory context. The PSE values (indicated by vertical dashed lines) were expressed as percentage of increase (1, 3, 6, 12, and 30%) or decrease (–1, –3, –6, –12, and –30%) in the length of the comparison relative to the reference interval. In this example, the PSE values of –5.6% measured with regular auditory context indicates that the comparison had to be 5.6% of 660 ms shorter than the reference, to be perceived as of equal length.

All auditory sequences comprised series of short sounds of 50 ms interspersed with blank intervals. Each sequence started with a series of low-frequency sounds of 440 Hz, randomly varying in number from 3 to 5, the “auditory context,” and was followed by two pairs of high-frequency sounds of 660 Hz. The time interval between the first pair of high-pitched sounds is referred to here as the “reference” interval, while the interval between the second pair is the “comparison” interval. The intervals between the low-frequency sounds in the auditory context could either be isochronous, with a constant duration of 660 ms, or centered on 660 ms and were varied randomly by adding a normally-distributed amount of jitter N (0, 100) ms. The duration of the auditory context was comparable between conditions. The reference interval lasted either 660, 462, or 330 ms. The duration of the comparison interval varied according to the method of constant stimuli: the comparison interval was expressed as a percentage of the reference interval and could take one of 10 values, with 5 increments and 5 decrements relative to the reference (±1, 3, 6, 12, and 30%). Each of the 10 comparison values was presented 10 times in each condition (**Figure 1**). Given that the length of the comparison was proportional to the length of the reference, the difference between reference and comparison intervals in terms of absolute duration was smaller for shorter intervals. According to the Weber–Fechner law (Fechner, 1860), the perceived change in a stimulus is proportional to the magnitude of the stimulus. Given that duration discrimination should be proportional to the duration of the reference interval, by adapting the length of the comparison to the length of the reference we expected to increase the sensitivity of our method and allow comparisons across intervals.

#### Design

**FIGURE 2:**
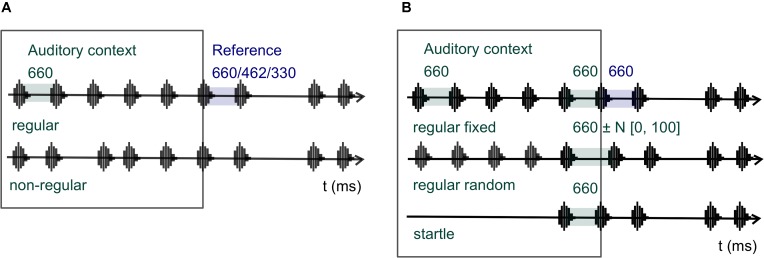
Stimuli of Experiment 1 **(A)** and Experiment 2 **(B)**: auditory context made of low-frequency sounds (regions surrounded by squares) and two pairs of high-pitched sounds delimiting the reference and the comparison intervals. The variables of Experiment 1 were auditory context (regular and non-regular) and Reference (660, 462, and 330 ms). In Experiment 2 only auditory context (regular fixed, regular random, and startle) was manipulated, while the duration of the reference interval was fixed to 660 ms.

**Figure 2A** illustrates the experimental design. The two factors manipulated were auditory context (regular and non-regular) and reference duration (660, 462, and 330 ms, corresponding to 100, 70, and 50% of the context interval). The regular auditory context was made of a series of sounds separated by intervals of constant duration; the non-regular context was made of a series of sounds separated by randomly varying time intervals. All participants completed all sessions, and the order of sessions and parts was counterbalanced across participants.

#### Procedure

Participants were seated at a table, wore headphones, and listened to sequences of short sounds. Their task was to compare the last two time intervals of each sequence - the ones interspersed between two high-pitched sounds - while ignoring the series of low-pitched sounds presented at the beginning of each trial. Participants were required to say whether the last interval, the comparison, was shorter or longer than the second-to-last interval, the reference. Participants answered by pressing one of two keys on a computer keyboard, and were instructed to respond as accurately as possible. No strict time limit was set for the response. A white noise sound of 100 ms signaled the beginning of each trial; the series of low- and high-frequency sounds began 600 ms later. Reference and comparison intervals were separated by a gap of 1200 ms. The experiment comprised three sessions, one for each reference (660, 462, and 330 ms). The sessions were run on separate days, not necessarily consecutive (mean ±*SD* = 4 ± 10 days in between). Each session was divided into two parts, one for each auditory context (regular and non-regular). Each part started with a practice block (20 trials) and included two experimental blocks (2 × 50 trials). The order of parts and sessions was counterbalanced.

#### Data Analysis

##### Pre-processing

The following criteria were set before starting data collection, to determine which data to include in the analysis: for each session, overall percentage correct ≥60%; mean percentage correct for the two easiest comparisons (±12%, 30%) ≥85%. Participants who performed below this cut-off in the first session were not asked to participate in the remaining sessions.

The data were fit with psychometric functions. The shape of the function was defined by the parameters “sigmoid” and “core,” which were set to “logistic” and “ab,” respectively^1^. The shape of the function was chosen amongst the many options provided by the toolbox Psignifit, so as to provide the best fit to our dataset. The Psignifit function “BootstrapInference” was used to find the best fit and to estimate the free parameters of the model, alpha and beta, while gamma and lambda were constrained to the range (0,0.06), to account for lapses in attention and errors unrelated to the stimulus properties, respectively. Alpha corresponds to the point of subjective equality (PSE), while beta is a measure of the spread of the function and is directly related to the just noticeable difference (JND). The dependent variables of interest in the analysis were the PSE and the JND. The PSE was calculated as the point on the curve corresponding to 50% of “longer” responses; PSE values indicate the amount of increase/decrease in the duration of the comparison interval relative to the reference interval that is necessary for the comparison to be perceived as equal to the reference. The JND was calculated as half of the difference between the 75% and 25% points. The PSE is a measure of bias, while the JND reflects precision.

The goodness of fit was assessed with the Psignifit function “GoodnessOfFit,” which returns a measure of the deviance of the data from the model and compares it against an empirical bootstrap distribution. A “good fit” was set as a further requirement for inclusion of the data in the analysis. The significance level of the test of “good fit” was set to 0.001, which corresponds to the value of 0.05 corrected with Bonferroni correction for 48 comparisons (24 participants, 2 conditions). We have chosen the conservative Bonferroni correction and adopted a strict further exclusion criterion, since we had already excluded during the course of the study all the participants unable to perform the task. Moreover, empirical bootstrap distributions were built out of small samples of 10 values; with small sample sizes, due to the limited variability in the sample, 95% confidence intervals contain the true parameter in less than 95% of the cases. In the analysis, whenever Mauchly’s test indicated violation of sphericity assumptions, Greenhouse-Geisser corrections were applied. Two-tailed paired Welch’s *t*-tests were applied in *post hoc* comparisons: the Welch’s *t*-test was preferred to the Student’s *t*-test, because it does not rely on the assumption of equality of variances, which is often violated in psychological experiments (Delacre et al., 2017). Bonferroni corrections were made to correct for multiple comparisons. The effect sizes reported are Cohen’s d (d) and generalized eta-squared (η_G_^2^). The data can be found on the Open Science Framework (OSF) repository^2^.

The first part of the analysis examined whether the perceived change in duration was proportional to the magnitude of the reference interval: PSE and JND values expressed in milliseconds were entered separately into a one-way repeated-measure ANOVA with reference (660, 462, and 330 ms) as the within-participant variable.

In the second part of the analysis, PSE and JND values expressed as percentages of the reference interval were entered into a two-way repeated-measures ANOVA with auditory context (regular and non-regular) and reference (660, 462, and 330 ms) as within-participant variables. PSE and JND were expressed as percentage of the reference interval, in order to average the values across reference intervals of different duration and compare the estimates between regular and non-regular auditory context.

In the third part, Bayesian analysis was applied to the non-significant comparisons of interest to our research question, in order to distinguish between evidence in favor of the null hypothesis from insensitive data (Dienes, 2014). A uniform distribution was chosen to represent the alternative hypothesis. The value chosen for the lower limit is standard and does not affect the results (Dienes, 2014), while the upper limit is set to the largest expected difference between the samples. Bayes factors for each comparison were obtained inserting the lower and the upper limits, the sample mean difference and the standard error of the difference in the online Bayes calculator provided by Zoltan Dienes^3^. Bayes factors <1/3 and >3 were interpreted as evidence for the null and the alternative hypotheses, respectively (see Dienes, 2014).

### Results

Four participants were excluded, because they did not meet the criterion set for the percentage correct (≥85%) for the two easiest conditions (S5: 72.5%; S6: 73.7%; S10: 83.7%; S18: 77.5%).

#### Duration Discrimination Across Reference Intervals – Absolute Measures

##### PSE

**FIGURE 3:**
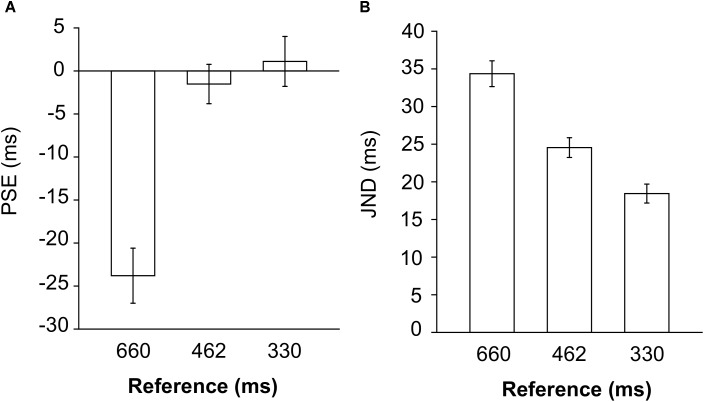
PSE **(A)** and JND **(B)** values expressed in milliseconds, measured for each reference interval, averaging the values across regular and non-regular auditory context.

The effect of reference was significant, *F*_(2,46)_ = 26.0, *p* = 3 × 10^-8^, η_G_^2^ = 0.30: the PSE values for the 660 ms reference [mean (*SD*) = -23.8 (24.3) ms] were different from the values for the 462 ms [mean (*SD*) = -1.52 (19.5) ms, *t*_(23)_ = 6.19, *p* = 3 × 10^-6^, *d* = 1.26] and the 330 ms reference [mean (*SD*) = 1.11 (10.8) ms, *t*_(23)_ = 5.64, *p* = 10^-5^, *d* = 1.15]; PSE values for the 462 ms and the 330 ms reference, instead, were comparable, *t*_(23)_ = 0.80, *p* = 0.43, *d* = 0.16. As shown in **Figure 3A**, PSE values for the 660 ms reference were negative and farther from zero, compared to the other intervals, indicating that in the former case, comparisons shorter than 660 ms were systematically judged as longer then the reference.

##### JND

The effect of reference was significant [*F*_(1.6,36.7)_ = 52.2, *p* = 2 × 10^-10^, η_G_^2^ = 0.40]: smaller JND values were observed for the 330 ms reference [mean (*SD*) = 18.4 (7.20) ms], compared to the 462 ms [mean (*SD*) = 24.5 (9.04) ms, *t*_(23)_ = 5.31, *p* = 2 × 10^-5^, *d* = 2.00] and the 660 ms reference [mean (*SD*) = 34.4 (13.0) ms, *t*_(23)_ = 9.81, *p* = 10^-9^, *d* = 1.49], as well as for the 462 ms [mean (*SD*) = 24.5 (9.04)], compared to the 660 ms reference [mean (*SD*) = 34.4 (13.0) ms], *t*_(23)_ = 5.27, *p* = 2 × 10^-5^, *d* = 1.08 (see **Figure 3B**). This result indicates that duration discrimination was more precise for shorter than longer reference intervals. An estimate of the Weber fraction is given by the ratio of the JND observed for each reference to the duration of the reference. In accordance with the Weber-Fechner law (Fechner, 1860), the Weber fractions for the 660 ms [Mean (*SD*) = 0.052 (0.015)], the 462 ms [Mean (*SD*) = 0.053 (0.017)] and the 330 ms reference [Mean (*SD*) = 0.056 (0.018)] were comparable (all *t*_(23)_ > 0.33).

#### Influence of a Regular Context on Duration Discrimination – Relative Measures

##### PSE

**FIGURE 4:**
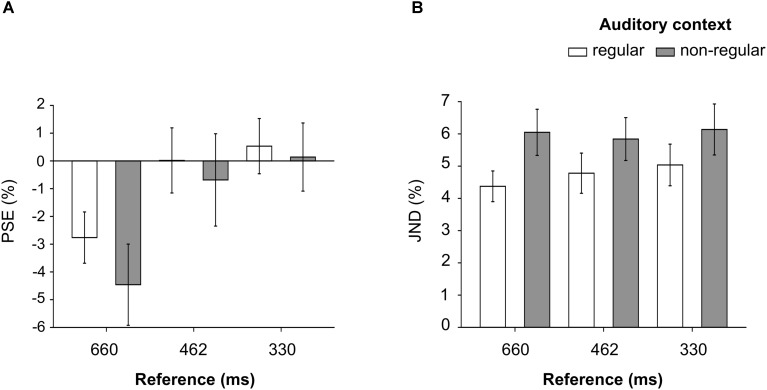
PSE **(A)** and JND **(B)** values expressed as a percentage of increase/decrease in the length of the comparison relative to the reference interval (%), measured for the 660 ms, the 462 ms and the 330 ms reference intervals, with regular and non-regular auditory context.

The main effect of reference was significant even when expressing the PSE as a percentage of increase/decrease in the length of the comparison relative to the reference [*F*_(2,46)_ = 16.6, *p* = 4 × 10^-6^, η_G_^2^ = 0.18]: once again, PSE values observed for the 660 ms reference [mean (*SD*) = -3.61 (3.68)%] were different from the PSE values for the 462 ms [mean (*SD*) = -0.33 (4.23)%, *t*_(23)_ = 5.03, *p* = 4 × 10^-5^, *d* = 1.03] and for the 330 ms reference [mean (*SD*) = 0.34 (3.28)%, *t*_(23)_ = 4.95, *p* = 5 × 10^-5^, *d* = 1.01], while the 462 ms and the 330 ms reference were comparable, *t*_(23)_ = 0.90, *p* = 0.40, *d* = 0.18. Neither the main effect of auditory context, *F*_(1,23)_ = 3.26, *p* = 0.08, η_G_^2^ = 0.016, nor the interaction between reference and auditory context, *F*_(2,46)_ = 2.37, *p* = 0.10, η_G_^2^ = 0.006, passed the threshold for significance testing (see **Figure 4A** and **Table 1**), indicating that the bias in duration discrimination, measured with different reference intervals was not modulated by auditory context.

**Table 1:** Means (standard deviations) of PSE and JND values, expressed as a percentage of the reference interval, for each condition of Experiment 1.

		PSE (%)		JND (%)	
	Auditory context	Regular	Non-regular	Regular	Non-regular
**Reference**	660	-2.76 (2.73)	-4.46 (4.33)	4.37 (1.41)	6.05 (2.12)
**(ms)**	462	0.02 (3.47)	-0.68 (4.92)	4.78 (1.84)	5.84 (1.97)
	330	0.53 (2.94)	0.14 (3.63)	5.04 (1.91)	6.14 (2.34)

##### JND

The effect of auditory context was significant [*F*_(1,23)_ = 28.8, *p* = 2 × 10^-5^, η_G_^2^ = 0.10], showing that overall the JND was smaller in the regular [mean (*SD*) = 28.9 (9.31)%] compared to the non-regular condition [mean (*SD*) = 39.8 (14.1)%]. This result indicates that duration discrimination was more precise when a regular, compared to a non-regular context preceded the intervals to be discriminated. The interaction between reference and auditory context, instead, did not pass the threshold for significance testing [*F*_(2,46)_ = 0.74, *p* = 0.48, η_G_^2^ = 0.005, see **Figure 4B** and **Table 1**], indicating that the difference in precision with regular compared to non-regular context was comparable across reference intervals.

#### Distinguishing Between No Evidence and Evidence for the Null With Bayes’ Factors

Bayesian analysis was applied to the interaction between auditory context and reference, to examine whether the effect of regular context observed for the JND was comparable across reference intervals (evidence for the null). The lower bound was set to 0%; the upper bound to 10.9%, the mean difference between the regular and the non-regular condition observed in Experiment 1. When we entered the values in the Bayes calculator we found evidence for the null, both when comparing the 660 ms and the 462 ms intervals [mean (*SE*) = 0.68 (0.62)%, *B* = 0.22], the 660 ms and the 330 ms intervals [mean (*SE*) = 0.71 (0.62)%, *B* = 0.25] and the 462 ms and the 330 ms intervals [mean (*SE*) = -0.03 (0.41)%, *B* = 0.04]. This result indicates that the difference in precision with regular compared to non-regular context was not modulated by the duration of the reference interval.

### Discussion

When the values were expressed in milliseconds, duration discrimination for the 660 ms reference was more biased, compared to the 462 ms and the 330 ms intervals, since in the former condition the comparison interval was systematically judged as shorter than the 660 ms reference, while discrimination with the other intervals was more veridical (**Figure 3A**).

Discrimination was also more precise for shorter compared to longer intervals, since smaller JND values were observed for the 330 ms reference, compared to all other intervals, and for the 462 ms compared to the 660 ms reference (**Figure 3B**). This decrease in precision with longer intervals can be explained by the Weber–Fechner law (Fechner, 1860), which states that the JND between two physical magnitudes is proportional to the absolute magnitude. As a result of this, the ratio of the JND to the absolute magnitude is constant (Weber fraction). In accordance with the Weber–Fechner law, the Weber fractions estimated for the 660 ms, the 462 ms and the 330 ms reference intervals were similar. These results justify our choice of adapting the length of the comparison interval to the length of the reference, so as to fine-tune our method. Besides, expressing PSE and JND values as percentages of the reference interval allowed us to compare the influence of the auditory context across reference intervals.

When the values were expressed as relative measures, the discrimination bias for the 660 ms reference was still present, but was not modulated by the characteristics of the auditory context, since neither the main effect of auditory context, nor the interaction between reference and auditory context passed the threshold for significance testing (**Figure 4A**). Nevertheless, this bias could have been introduced by the auditory context, since both in the regular and non-regular conditions context intervals were close to 660 ms in duration. Since we have not come across a similar study reporting a similar perceptual bias, we have decided to refrain from speculation about this unexpected effect.

Duration discrimination, instead, was more precise with regular, compared to non-regular context, since smaller JND were observed when a regular context was presented. Contrary to expectations (i.e., Barnes and Jones, 2000), however, this effect of regular context was not modulated by the duration of the reference interval. The DAT, instead, would predict better discrimination when the reference interval is equal in length to the intervals in the regular context, compared to shorter and longer intervals. In their study, Barnes and Jones (2000) observed that discrimination, measured as proportion correct, was best when the first interval to be discriminated lasted as long as the intervals in the regular auditory context, while it progressively decreased for longer and shorter intervals. Our data do not strongly support the DAT, given that we found evidence for the null, when we compared the effect of context regularity observed for the JND across reference intervals.

Regarding the main effect of auditory context observed for the JND, at least two factors can account for the difference in performance with regular and non-regular context: the easiness with which the beginning of the task can be anticipated, and the influence of periodic auditory stimulation on attention. In Experiment 1, we tried to make the reference interval unexpected, by presenting it after a variable number of low-frequency sounds, chosen at random between 3 and 5. Despite this attempt, the first high-pitched sound marking the reference interval in the regular, unlike in the non-regular condition, was always aligned with the low-pitched sounds in the context. The advantage observed with a regular context could therefore reflect the ability of participants to anticipate the task, rather than being the result of an entrainment between attention and the ongoing periodicity.

A second experiment was carried out with the intent to isolate the factors of anticipation and background regularity, and compare their individual contributions to interval discrimination against a condition in which both factors are at play. The objective of Experiment 2 was to examine how likely each factor contributes to the effect of regular context observed in Experiment 1.

## Experiment 2

### Materials and Methods

#### Participants

Sixteen participants were recruited. Four were removed (see below), leaving a sample of 12 included in the study (mean ±*SD* age = 23.2 ± 3.3 years, 8 females). Participants provided written informed consent and participated in the experiment in return for payment (£7). All participants had normal hearing and no history of audiological or neurological disorder. The study was approved by the research ethics committee of the University of Nottingham (SoPEC 932) and was conducted in accordance with the Declaration of Helsinki (as of 2008).

#### Stimuli

The stimuli of Experiment 2 were similar to the stimuli used in Experiment 1. The number of sounds in the auditory context varied between conditions and was either randomly selected between 3, 4, and 5, similarly as in Experiment 1, or it was equal to 1. When more than one low-frequency sound was presented, all sounds were regularly spaced, separated by a constant interval of 660 ms. The duration of the interval preceding the first high-pitched sound was either 660 ms, or centered around 660 ms and were varied randomly by adding a normally-distributed amount of jitter N (0, 100) ms. The duration of the reference interval was fixed at 660 ms.

#### Design

**Figure 2B** illustrates the design of Experiment 2. Auditory context is the single variable manipulated: the regular fixed context was a series of sounds randomly varying in number from 3 to 5, separated by blank intervals of 660 ms; the startle context was a single low-frequency sound, preceded and followed by blank intervals, the first varying in duration and the second fixed to 660 ms; the regular random context was the same as regular fixed, except for the fact that the duration of the interval preceding the first high-pitched sound was centered on 660 ms and varied randomly.

Despite the fact that the duration of the auditory context was comparable between conditions, the point in time when the reference was presented was not equally predictable. The reference could be anticipated easily in the startle condition, given that the first high-pitched sound always followed the single low-frequency sound by 660 ms. The regular fixed context was intermediate in terms of expectancy, as the first high-frequency sound was always aligned with the regular context, and was always presented after either 3, 4, or 5 low-frequency sounds. Regular random was the least predictable condition, despite being composed of regularly spaced sounds, because the length of the interval preceding the reference was varied randomly from trial to trial.

#### Procedure

Task and setting were the same as in Experiment 1. Experiment 2 comprised three parts, one for each level of auditory context (regular fixed, regular random, and startle), run consecutively in a single session. Each part started with a practice block (20 trials) and was followed by two experimental blocks (2 × 50 trials). The order of the parts was counterbalanced.

#### Analysis

In the first part of the analysis, PSE and JND values were entered separately in a one-way repeated-measures ANOVA with auditory context (regular fixed, regular random, and startle) as within-participant variable.

In the second part, Bayesian analysis was applied in order to discriminate between insensitive data and evidence for the null. Similarly as in Experiment 1, the alternative hypothesis was represented with a uniform distribution with the lower limit fixed to a standard and the upper limit set to the mean difference between the regular and non-regular conditions measured in Experiment 1 for the 660 ms reference, which corresponds to the largest difference we expected to observe for the effect of auditory context (**Table 2**).

**Table 2:** Means (standard deviations) in milliseconds for PSE and JND values, measured in Experiments 1 and 2 for the 660 ms reference interval, with different auditory context.

Experiment	Auditory context	PSE (ms)	JND (ms)
E1	Regular	-18.2 (18.0)	28.9 (9.31)
E1	Non-regular	-29.4 (28.6)	39.8 (14.1)
E2	Regular fixed	-13.5 (24.2)	30.0 (11.8)
E2	Regular random	-14.9 (29.2)	32.7 (12.2)
E2	Startle	-12.2 (30.2)	33.6 (13.0)

### Results

Four participants were excluded from the analysis: three participants were excluded because they did not meet the criterion set for percentage correct (≥85%) for the two easiest conditions (S3: 80.5%; S9: 73.7%; S13: 83.7%); one participant was excluded due to loss of data.

#### PSE

**FIGURE 5:**
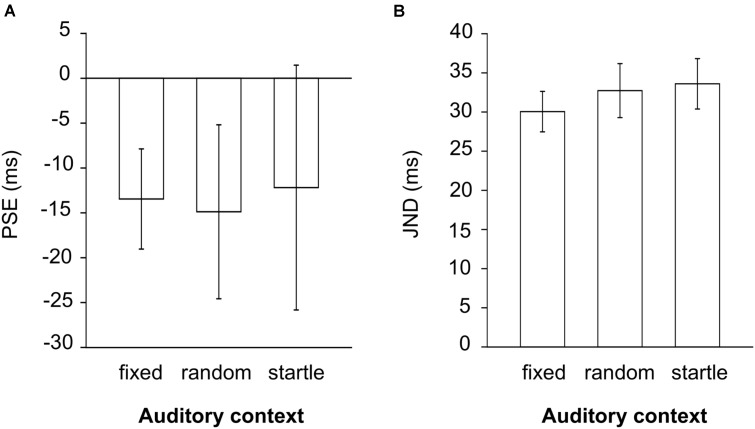
PSE **(A)** and JND **(B)** values expressed in milliseconds, measured with regular fixed (fixed), random regular (random), and startle auditory context in Experiment 2.

The main effect of auditory context for PSE was non-significant [*F*_(1.1,12.6)_ = 0.034, *p* = 0.89, η_G_^2^ = 0.002, **Figure 5A**]. Since a difference in bias with regular compared to non-regular context was not present in Experiment 1, this effect was not examined further with Bayesian analysis. Similarly, as in Experiment 1, negative mean PSE values were observed for all the conditions.

#### JND

The main effect of auditory context for JND was also non-significant [F_(2,22)_ = 0.68, *p* = 0.52, η_G_^2^ = 0.016, **Figure 5B**]. When we applied Bayesian analysis (lower bound = 0 ms, upper bound = 10.8 ms) we found that the data was insensitive and did not support the null, both when comparing regular fixed with regular random [mean (*SE*) = 2.68 (2.64) ms, *B* = 0.87], regular fixed with startle [mean (*SE*) = 3.55 (2.42) ms, *B* = 1.56] and regular random with startle [mean (*SE*) = 0.87 (2.39) ms, *B* = 0.46]. The values observed for regular fixed, regular random, and startle auditory context in Experiment 2 were closer to the values measured with regular, compared to non-regular context in Experiment 1 (**Table 2**).

### Discussion

Duration discrimination both in terms of bias and precision, did not differ between regular fixed, regular random, and startle auditory context. Given that the ANOVAs on the PSE and the JND values were not significant and that Bayesian analysis did not provide evidence in favor of the null, no conclusion can be drawn from this second study.

## General Discussion

In our work, we examined whether a regular compared to a non-regular context can influence duration perception, measured with a 2-IFC perceptual judgment task. Our study was based on the work of Barnes and Jones (2000), who had previously shown that a regular context systematically influence the perceived duration of single auditory events, embedded in this context. In a series of seven experiments, these authors showed that listeners were more accurate in judging the duration of intervals that were equal in length to the intervals presented in the regular context, compared to shorter or longer intervals. In describing their results, the authors postulated that attention fluctuates over time and synchronizes with the ongoing auditory periodicity.

In our study, differently from Barnes and Jones (2000), we introduced a non-regular condition and used PSE and JND values to measure duration discrimination, instead of percentage correct. Our work aimed at measuring a general effect of regular compared to non-regular context; it also examined the effect of context regularity across reference intervals of different duration. Despite these differences, we expected to support the finding of Barnes and Jones (2000) by showing that duration discrimination with regular compared to non-regular context, was significantly better when the reference interval was the same as the context intervals (660 ms reference), compared to shorter intervals (462 and 330 ms). Even though temporal discrimination was more precise with regular compared to non-regular context, this effect was not selective for the duration of the reference interval. Bayesian analysis on the non-significant interaction between reference and auditory context in Experiment 1 provided evidence for the null hypothesis and supported the conclusion that the effect of context regularity was not modulated by the duration of the reference interval. Further, duration discrimination for the 660 ms reference interval was also more biased, compared to the other reference intervals, but this effect was not modulated by the characteristics of the auditory context.

Our speculation is that the divergence between our results and the results of Barnes and Jones (2000) stems from the methods used to quantify discrimination performance and from the range of reference intervals included in the experimental blocks.

First, Barnes and Jones (2000) assumed a constant linear relation between the perceived change in duration and the duration of the reference interval, and applied the Weber fraction to set the duration of the comparison interval. The comparison interval was expressed as a constant proportion of the reference interval and was either 0.12 (experiments from 1 to 6) or 0.09 (experiment 7). In our design the duration of the comparison interval was also proportional to the reference interval, but instead of being fixed, it varied among five magnitudes. Having a single magnitude for the comparison may not be ideal when comparing discrimination across reference intervals. Applying the Weber fraction, in fact, assumes a perfect linear relation between perceived change and magnitude of the stimulus; while a linear relation may hold for small magnitudes, a non-linear relation is more likely to apply to the discrimination of larger magnitudes [e.g., logarithmic, Weber–Fechner law (Fechner, 1860)].

Second, while Barnes and Jones presented trials with different reference intervals within the same block, we had a separate block for each reference (660, 462, and 330 ms). In three of their experiments (experiments 4, 5, and 6), Barnes and Jones (2000) included a no-context condition as a negative control. It is worth noting that even for this control condition they found an expectancy profile very similar to the one observed when a temporal auditory context preceded the temporal judgment. In their paper, the authors attributed this unexpected finding to the “session range effect” (p. 283); explaining that judgments made about the interval duration are influenced by the range of values that occur in a block or session (Woodrow, 1951; Jamieson and Petrusic, 1975; Hellstrom, 1977, 1985; Allan, 1979). Since the expectancy profile found with auditory context was significantly greater than the profile found with no-context, Barnes and Jones attributed the expectancy profile to the influence exerted by contextual information on discrimination performance. In order to prove the robustness of this effect of context regularity, however, the results should be free from confounds: if the characteristic U-shaped expectancy profile results from presenting a regular auditory context prior to the discrimination, it should be observed independently of the range of values presented in each block or session.

Other studies, in fact, have shown that perceptual judgment can be affected by the temporal context generated over the course of an experiment (Jazayeri and Shadlen, 2010; Cicchini et al., 2012). Jazayeri and Shadlen (2010) asked participants to reproduce the duration of intervals delimited by pairs of flashes, where these intervals were extracted from different probability distributions. The authors observed that participants’ responses tended to gravitate toward the mean of the distribution from which the intervals were extracted, overestimating the duration of shorter intervals and underestimating the duration of longer intervals. Cicchini et al. (2012), however, showed that this central tendency effect, both in non-musicians and bowstring musicians, was less evident when the intervals were delimited by sounds, rather than by flashes, indicating that duration reproduction was more veridical in the auditory modality, compared to the visual modality. Furthermore, the authors did not observe a central tendency effect in drummers, which suggests that the influence of prior experience on perceptual judgment may be related to the degree of uncertainty in the response.

In a related line of study, Rhodes and Di Luca (2016) have shown that the perceived regularity of a sequence of sounds is influenced by the degree of regularity of the environment in which the sequence is embedded, thus suggesting that perception of temporal regularities is also affected by expectation.

## Conclusion

In our work we re-examined the effect of a regular auditory context on duration discrimination, previously shown by Barnes and Jones (2000) and part of the DAT. According to DAT, duration discrimination benefits maximally from a regular context when the reference interval, the first interval to be discriminated, is equal in duration to the intervals in the context. Discrimination of intervals shorter or longer than the context interval, instead, progressively decreases.

With our experiments we showed that duration discrimination of two intervals presented in a sequence can be influenced by an auditory context, since discrimination in Experiment 1 was more precise when reference and comparison intervals were preceded by a regular compared to a non-regular context. Contrary to what we expected in view of Barnes and Jones (2000), however, this difference in discrimination with regular compared to non-regular context did not depend on the relationship among the intervals in the context and the first interval to be estimated. Not only was discrimination with regular compared to non-regular context not selective for the duration of the reference interval, but discrimination for the 660 ms reference interval was also more biased compared to the 462 and 330 ms intervals, both with PSE values expressed in absolute and in relative measures. According to our understanding, the discrepancy between our results and those of Barnes and Jones (2000) is likely to be due to the differences in the methods and measurements used to quantify discrimination performance and to the presence of the “session range effect,” likely a confounder, in Barnes and Jones (2000).

## Author Contributions

SZ designed the experiment, collected and analyzed the data, and wrote the manuscript. NH commented and edited the manuscript.

## Conflict of Interest Statement

The authors declare that the research was conducted in the absence of any commercial or financial relationships that could be construed as a potential conflict of interest.

## Footnotes

1 http://psignifit.sourceforge.net/WELCOME.html

2 osf.io/wm3cs

3 http://www.lifesci.sussex.ac.uk/home/Zoltan_Dienes/inference/bayes_factor.swf

## References

[B1] Adams R. D. (1977). Intervening stimulus effects on category judgments of duration. *Percept. Psychophys.* 21 527–534. 10.3758/BF03198733

[B2] Allan L. G. (1979). The perception of time. *Percept. Psychophys.* 26 340–354. 10.3758/BF03204158

[B3] Barnes R. Jones M. R. (2000). Expectancy, attention, and time. *Cogn. Psychol.* 41 254–311. 10.1006/cogp.2000.0738 11032658

[B4] Brainard D. H. (1997). The psychophysics toolbox. *Spat. Vis.* 10 443–446. 10.1163/156856897X00357 9176952

[B5] Buffardi L. (1971). Factors affecting the filled-duration illusion in the auditory, tactual, and visual modalities. *Percept. Psychophys.* 10 292–294. 10.3758/BF03212828

[B6] Buhusi C. V. Meck W. H. (2005). What makes us tick? Functional and neural mechanisms of interval timing. *Nat. Rev. Neurosci.* 6 755–765. 10.1038/nrn1764 16163383

[B7] Burr D. Della Rocca E. Rocca E. Morrone M. (2013). Contextual effects in interval-duration judgements in vision, audition and touch. *Exp. Brain Res.* 230 87–98. 10.1007/s00221-013-3632-z 23864044

[B8] Cicchini G. Arrighi R. Cecchetti L. Giusti M. Burr D. (2012). Optimal encoding of interval timing in expert percussionists. *J. Neurosci.* 32 1056–1060. 10.1523/JNEUROSCI.3411-11.2012 22262903 PMC6621155

[B9] Delacre M. Lakens D. Leys C. (2017). Why psychologists should by default use Welch’s T-test instead of student’s t-test. *Int. Rev. Soc. Psychol.* 30 92–101. 10.5334/irsp.82

[B10] Dienes Z. (2014). Using Bayes to get the most out of non-significant results. *Front. Psychol.* 5:781. 10.3389/fpsyg.2014.00781 25120503 PMC4114196

[B11] Drake C. Botte M. C. (1993). Tempo sensitivity in auditory sequences: evidence for a multiple-look model. *Percept. Psychophys.* 54 277–286. 10.3758/BF03205262 8414886

[B12] Fechner G. (1860). *Elemente der Psychophysik.* Wiesbaden: Breitkopf und Härtel.

[B13] Fründ I. Haenel N.V. Wichmann F.A. (2011). Inference for psychometric functions in the presence of nonstationary behavior. *J. Vis.* 11:16. 10.1167/11.6.16 21606382

[B14] Hellstrom A. (1977). Time errors are perceptual: an experimental investigation of duration and a quantitative successive-comparison model. *Psychol. Res.* 39 345–388. 10.1007/BF00308933

[B15] Hellstrom A. (1985). The time-order error and its relatives: mirrors of cognitive processes in comparing. *Psychol. Bull.* 97 35–61. 10.1037/0033-2909.97.1.35

[B16] Horr N. K. Di Luca M. (2015). Taking a long look at isochrony: perceived duration increases with temporal, but not stimulus regularity. *Atten. Percept. Psychophys.* 77 592–602. 10.3758/s13414-014-0787-z 25341650 PMC4335101

[B17] Jamieson D. G. Petrusic W. M. (1975). The dependence of time-order error direction on stimulus range. *Can. J. Psychol.* 29 175–182. 10.1037/h0082023 1175095

[B18] Jazayeri M. Shadlen M. N. (2010). Temporal context calibrates interval timing. *Nat. Neurosci.* 13 1020–1026. 10.1038/nn.2590 20581842 PMC2916084

[B19] Jones M. Moynihan H. MacKenzie N. Puente J. (2002). Temporal aspects of stimulus-driven attending in dynamic arrays. *Psychol. Sci.* 13 313–319. 10.1111/1467-9280.00458 12137133

[B20] Karmarkar U. R. Buonomano D. V. (2007). Timing in the absence of clocks: encoding time in neural network states. *Neuron* 53 427–438. 10.1016/j.neuron.2007.01.006 17270738 PMC1857310

[B21] Large E. W. Jones M. R. (1999). The dynamics of attending: how people track time-varying events. *Psychol. Rev.* 106 119–159. 10.1037/0033-295X.106.1.119

[B22] McAuley J. D. Kidd G. R. (1998). Effect of deviations from temporal expectations on tempo discrimination of isochronous tone sequences. *J. Exp. Psychol. Hum. Percept. Perform.* 24 1786–1800. 10.1037/0096-1523.24.6.1786 9861723

[B23] Pelli D. G. (1997). The video toolbox software for visual psychophysics: transforming numbers into movies. *Spat. Vis.* 10 437–442. 10.1163/156856897X00366 9176953

[B24] R Core Team (2016). *R: A Language and Environment for Statistical Computing.* Vienna: R Foundation for Statistical Computing.

[B25] Rammsayer T. H. Leutner D. (1996). Temporal discrimination as a function of marker duration. *Percept. Psychophys.* 58 1213–1223. 10.3758/BF03207554 8961832

[B26] Rammsayer T. H. Lima S. D. (1991). Duration discrimination of filled and empty auditory intervals: cognitive and perceptual factors. *Percept. Psychophys.* 50 565–574. 10.3758/BF03207541 1780204

[B27] Rhodes D. Di Luca M. (2016). Temporal regularity of the environment drives time perception. *PLoS One* 11:E0159842. 10.1371/journal.pone.0159842 27441686 PMC4956244

[B28] Spencer R. Karmarkar U. Ivry R. (2009). Evaluating dedicated and intrinsic models of temporal encoding by varying context. *Philos. Trans. R. Soc. B Biol. Sci.* 364 1853–1863. 10.1098/rstb.2009.0024 19487188 PMC2685823

[B29] Thomas E. C. Brown I. (1974). Time perception and the filled- duration illusion. *Percept. Psychophys.* 16 449–458. 10.3758/BF03198571

[B30] Woodrow H. (1951). “Time perception,” in Handbook of experimental psychology, ed. Langfeld H. S. (New York, NY: Wiley). 1224–1236.

