# Corrigendum: The Effect of a Regular Auditory Context on Perceived Interval Duration

**DOI:** 10.3389/fpsyg.2018.02157

**Published:** 2018-11-07

**Authors:** Silvia Zeni, Nicholas P. Holmes

**Affiliations:** School of Psychology, University of Nottingham, Nottingham, United Kingdom

**Keywords:** duration perception, time perception, rhythm, temporal regularity, audition, attention

In the original article, there was an error in the funding statement. The funding statement in the original article was not formatted according to the funder requirements. It appeared in the original version as: This research was supported by the Medical Research Council (grant number UKP4200 to SZ). It should have been formatted as: This work was supported by the Medical Research Council [grant number UKP4200] to SZ. The authors apologize for this error and state that this does not change the scientific conclusions of the article in any way. The original article has been updated.

## Conflict of interest statement

The authors declare that the research was conducted in the absence of any commercial or financial relationships that could be construed as a potential conflict of interest.

